# Carbonate-based Janus micromotors moving in ultra-light acidic environment generated by HeLa cells *in situ*

**DOI:** 10.1038/srep21701

**Published:** 2016-02-24

**Authors:** Maria Guix, Anne K. Meyer, Britta Koch, Oliver G. Schmidt

**Affiliations:** 1 Institute for Integrative Nanosciences, Leibniz Institute for Solid State and Materials Research Dresden, 01069 Dresden, Germany; 2 Material Systems for Nanoelectronics, Chemnitz University of Technology, 09126 Chemnitz, Germany; 3 Center for Advancing Electronics Dresden, Dresden University of Technology, 01187 Dresden, Germany

## Abstract

Novel approaches to develop naturally-induced drug delivery in tumor environments in a deterministic and controlled manner have become of growing interest in recent years. Different polymeric-based microstructures and other biocompatible substances have been studied taking advantage of lactic acidosis phenomena in tumor cells, which decrease the tumor extracellular pH down to 6.8. Micromotors have recently demonstrated a high performance in living systems, revealing autonomous movement in the acidic environment of the stomach or moving inside living cells by using acoustic waves, opening the doors for implementation of such smart microengines into living entities. The need to develop biocompatible motors which are driven by natural fuel sources inherently created in biological systems has thus become of crucial importance. As a proof of principle, we here demonstrate calcium carbonate Janus particles moving in extremely light acidic environments (pH 6.5), whose motion is induced in conditioned acidic medium generated by HeLa cells *in situ*. Our system not only obviates the need for an external fuel, but also presents a selective activation of the micromotors which promotes their motion and consequent dissolution in presence of a quickly propagating cell source (i.e. tumor cells), therefore inspiring new micromotor configurations for potential drug delivery systems.

The development of autonomous nano- and micromotors has been of growing interest due to potential applications in both biomedical^1^ and environmental fields^2^, starting from the well-known catalytic motors to the ones driven by an external source (e.g. magnetic field, light, electric field, ultrasonic acoustic waves)^3^. Studies of the initial micromotor configurations, mainly based on microstructures whose catalytic platinum surface is activated in presence of hydrogen peroxide (H_2_O_2_), have led to a better understanding of motion principles at low Reynolds numbers and the feasibility of using such entities in complex natural environments^4^,^5^,^6^,^7^,^8^. Major steps forward in the field of catalytic micromotors partly rely on the fine tuning of the motors’ movement not only by using a magnetic field^9^,^10^,^11^, but also by employing light illumination^12^ or thermal effects^13^,^14^ to start and stop the engines. Alternative approaches focused on using external energy sources to promote fuel-free operation of the motors, such as acoustic^15^,^16^,^17^ or magnetic^18^,^19^,^20^ energy, leading to remotely controlled non-invasive systems for future *in vivo* applications, such as microsurgery^21^,^22^. Additionally, it is worth to mention the cooperative behaviour and self-assembly of micromotors^23^,^24^, along with thermo and phototactic-related research^25^,^26^, all of key importance towards the development of complex organized systems where situations taking place in living environments are mimicked.

One of the most recent trends in the field of catalytic micromotors is focused on the development of autonomous entities which are driven by biocompatible fuels, ideally present in the same environment where the reaction is aimed to take place. Microtubular motors based on a catalytic inner zinc layer were reported to move in extremely acidic mouse stomach in an *in vivo* study, performing an autonomous release of the cargo on the stomach wall after its gradual dissolution in the gastric environment^27^. Alternative non-catalytic Ru/Au bimetallic nanomotors were recently reported to be controlled inside living cells by using acoustic waves^28^.

Among the potential biomedical applications where nanomaterials and nanotechnology could play a major role, imaging techniques for early cancer detection and related treatment therapies are on the priority list. Although He *et al.* reported the use of tubular polymer-based modified with Pt nanoparticles whose motion was related to a spontaneous photothermal effect^13^ and Janus nanomotors based on mesoporous silica nanoparticles with Cr/Pt metallic caps for drug release^29^, the future implementation of such micromotors is still hindered by requiring H_2_O_2_ as fuel (toxic to mammalian cellular function). However, a recent approach reported by Wilson *et al.* demonstrated the directional and autonomous movement of polymer stomatocytes loaded with platinum nanoparticles towards H_2_O_2_ secreting neutrophil cells, showing its potential as emerging carriers for drug delivery^30^. Regarding photothermal cancer treatment, multifunctional nanoparticles have arisen as potential artefacts to selectively enhance the efficacy of photothermal ablation therapy. Apart from the possibility to deliver both heat and drug to tumorigenic regions resulting in a more selective and effective treatment with minimal side effects, it is of special interest that they are biocompatible and biodegradable^31^,^32^.

The present work is inspired by the use of well-known effervescent systems based on carbonate materials (e.g. sodium carbonate, calcium carbonate) that permit the enhanced dissolution of the active pharmaceutical compound of interest, which is generally plagued by a low dissolution rate^33^. To promote the activation of such an effervescent system an acidic environment is required. Such biocompatible tablets are not only present in our daily life (i.e. Aspirin, Ibuprofene, Vitamin C), but are potentially interesting for nano and micrometric platforms for environmental and drug delivery/biosensing applications^34^. The easy-loading and functionalization of such carbonate based systems, along with pH-sensitive dissolution, make them the perfect carriers for cancer cell treatment.

The production of lactic acid as a by-product of anaerobic glucose metabolism in tumour tissues induces acidosis^35^, showing an average extracellular pH value of 6.5 and even reaching lower pH values from 5.0 to 5.5 in intracellular components (e.g. endosomes, lysosomes)^36^. Such pH gradients have been exploited for the conception and development of pH-responsive nanomaterials that can release bioactive compounds without any external trigger^37^. Carbonate systems have been of particular interest for being biodegradable, nontoxic to normal cells and present a cumulative drug release in acid-dependent systems^38^ along with their use as carriers to encapsulate and adsorb bioactive compounds^39^,^40^. Other interesting features have been observed for calcium carbonate particles, such as their application as micropumps by using differently charged particles as tracers to show the diffusioosmotic flows arising in an unsaturated aqueous solution^41^.

Carbonate-based microstructures were successfully applied for cargo loading and delivery in presence of acidosis metabolism^38^, encouraging us to exploit such material as biocompatible micromotors with the potential for directed drug delivery. Here we implement this concept by the synthesis and characterization of novel biocompatible and biodegradable asymmetric Janus motors, which move in the presence of extremely light acidic conditions attributed to tumorous growth (see Fig. 1c). We analyzed their motion patterns to determine the mechanism behind their movement and present their dissolution and movement in acidic environments that were produced *in situ* by Hela cells.

## Results

### Synthesis and characterization of calcium carbonate Janus particles (CaCO_3_ Janus particles)

**Figure 1:**
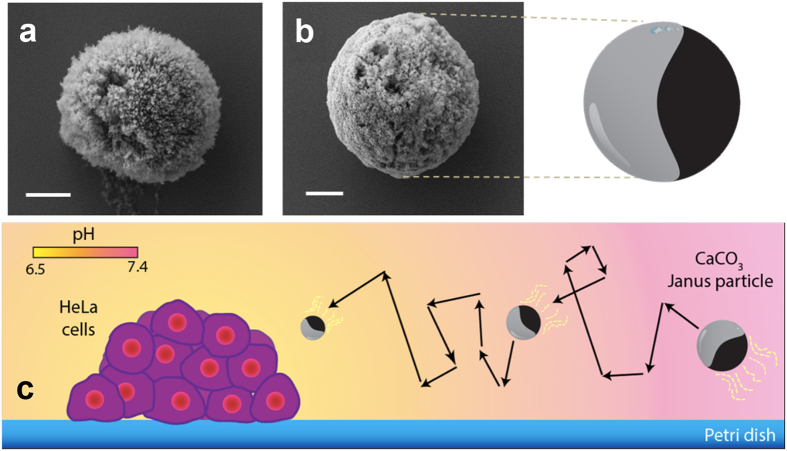
Morphological characterization of CaCO_3_ Janus particles. SEM images of bare CaCO_3_ (**a**) and CaCO_3_ Janus particles, with a half of its surface covered with cobalt thin layer, along with the schematic from the CaCO_3_ Janus particles (**b**). Schematic of CaCO_3_ particles moving in acidic environment *in situ* generated by HeLa cells (**c**). Scale bar = 2 μm.

Calcium carbonate microparticles were synthesized under sonication mixing conditions and conveniently dispersed onto glass substrates to further deposit a 0.8nm layer of cobalt. Such a cap induces structural asymmetry, a key feature for the directional motion of Janus particles. The present cap composition served for asymmetry issues, not showing any toxicity for short-term experiments. However, for long term experiments in future biomedical applications alternative cap compositions should be considered. Regarding characterization of the Janus particles, all the studies were performed before the cobalt cap was depositied onto the microstructures. The SEM study reveals a clearly porous surface of the particles and a size range of 6.64 ± 3.42 μm (see Fig. 1a). The crystalline structure of the particles was evaluated by XRD studies, where vaterite and calcite phases are identified in the corresponding XRD pattern (see Figure S2). The composition of calcium carbonate in the microparticles was obtained by SEM-EDX, showing the expected weight percentage per each element (see Figure S3), in addition to the SEM-EDX from the asymmetric CaCO_3_ Janus microparticles (see Figure S4).

### Motion principle under controlled acidic pH conditions

Carbonate structures are generally associated to effervescent systems, which are directly related to the liberation of carbon dioxide (CO_2_). Indeed, the essential elements in such effervescent tablets where the *in situ* creation of carbon dioxide takes place are a mixture of acids (e.g. citric acid and tartaric acid), a CO_2_ donor, which corresponds to the carbonate structure (e.g. sodium bicarbonate, calcium carbonate) and the active additive (e.g. analgesics, antibiotics). All ingredients undergo a process of wet granulation, dry granulation and fusion, which is further moistened with water to obtain the effective dissolution of the drug, rapidly disintegrating and quickly releasing the active additive in less than 12 seconds. However, in our specific application, the motion principle in tumor cell conditioned acidic environment showed no bubble generation. This perfectly suited our further experimental conditions, as our interest was not focused on massively produced bubble bursts as they would not be recommendable for future *in vivo* applications.

**Figure 2:**
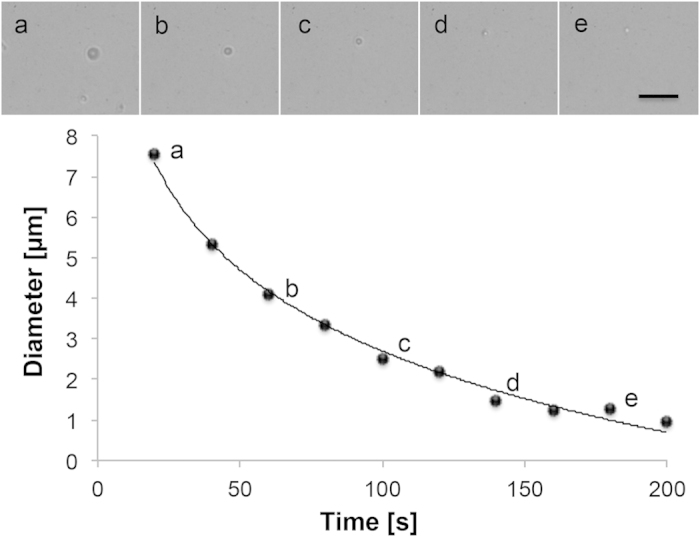
Dissolution trend during CaCO_3_ Janus particles motion. Size vs. time of CaCO_3_ particles at a Mannitol:Sodium dihydrogen citrate 2.5·10^−9^ : 1.25·10^−9^ M concentration. Scale bar, 25 μm.

Additionally, the scope of our study was taking advantage of the acidic conditions arising from acidosis metabolism. Both conditions, the lack of massive bubble production and working in light acidic environments, defined our experimental studies. Therefore, they were held in presence of a well-known and very low acidic concentration range, as to come close to the final acidic conditions naturally induced by Hela cells. CaCO_3_ Janus particles were tested in parafilm platforms as to avoid fluxes during the experiments in presence of a 2.5:1.25 nM Mannitol:dihydrogen citrate solution which leads to controlled acidic conditions (see Movie S1). This combination of acids is reportedly used at higher concentration for the effervescent formulation of ibuprofen^42^. The pH value of the resulting solution was 4.6, which was enough to induce a non-Brownian motion of CaCO_3_ Janus particles associated with a controlled dissolution of the CaCO_3_ structure. An exponential decay of the particles’ diameter was clearly observed during motion (see Fig. 2). Different concentrations were tested in the nanomolar range, showing that the higher the acid concentration was, the faster its dissolution and therefore its related motion was (see SI Fig. 1), reaching velocities of 1.8 μm/s.

Although this speed is not comparable to other Pt-based catalytic motors, it should be noted that here we report by far the lowest acidic concentration (2.5:1.25 nM Mannitol:dihydrogen citrate solution compared to the reported 0.1 M in PANI/Zn micromotors^43^) capable of driving a single micromotor. Viability studies in controlled acidic conditions for both, HeLa cells and Fibroblasts, revealed that neither HeLa cells nor Fibroblasts were affected in cell survival by the presence of the CaCO_3_ Janus particles or their ions after being dissolved (see Figure S6).

The key features associated with the motion of the CaCO_3_ Janus particles are the break of symmetry (conferred by the cobalt cap) and the local chemical gradients created by the controlled decomposition of the calcium carbonate in presence of acids, giving rise to Ca^2+^, HCO_3_^−^ (which evolve to CO_2_), OH^−^ and H^+^ ions (see SI Movie 2). While Sen *et al.* described calcium carbonate particles as potential micropumps by taking advantage of the diffusioosmosis processes in unsaturated solutions related to different diffusion coefficients of the generated ions^41^, we here use diffusioosmotic flows to induce the motion of our asymmetric calcium carbonate Janus microparticles in acidic pH conditions.

### Tumour cell conditioned acidic environment as potential fuel for micromotors

**Figure 3:**
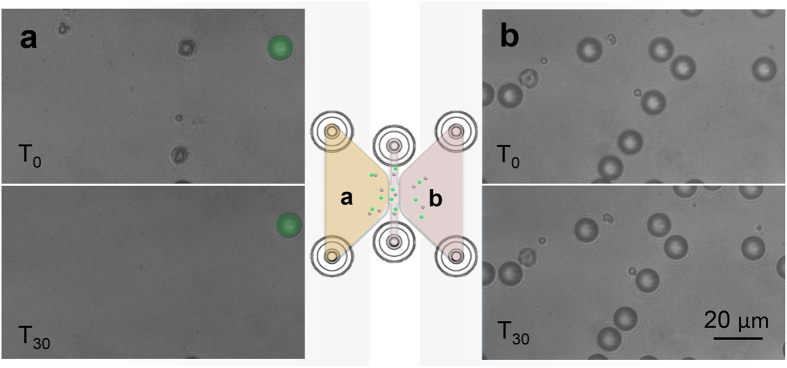
Acidic medium *vs* non-buffered medium. Optical image of CaCO_3_ Janus particles and fluorescent latex reference particles in presence of conditioned media (**a**) at time 0 and after 30 minutes (reference particles highlighted in green), and in presence of non-buffered media (**b**) at time 0 and after 30 minutes. Schematic of the IBIDI chip, depicting CaCO_3_ Janus particles and reference particles in the middle channel, which are also dispersed in the chamber filled up with conditioned media (**a**) and the chamber filled up with non-buffered media (**b**). Scale bar, 20 μm.

Once the directional motion of CaCO_3_ Janus particles was observed at nanomolar acidic range, further studies focused on implementing conditioned tumour cell media as physiologically, or rather pathologically, present fuel. Experiments were carried out on IBIDI chips (IBIDI GmbH, Munich, Germany), conceived as ideal platforms for long-term chemotaxis studies^44^. Such microfluidic chips are based on two separated chambers connected with a middle channel, which permits the evaluation of the motion of the Janus particles under closed and sterile conditions in absence of any flow (see Figure S5b). We applied them to study the controlled dissolution and related motion of CaCO_3_ particles in conditioned HeLa cell medium in one chamber with direct control experiments in presence of non-conditioned, non-buffered cell medium in the other chamber. Therefore, one of the chambers was filled with conditioned HeLa cell media (pH 6.5), while the other was filled with non-conditioned, non-buffered cell medium (pH 7.4) as a negative control. Janus and reference particles of similar size were introduced in the middle chamber being distributed not only in the middle channel but also in each chamber (see Fig. 3). The motion of the particles was evaluated in both chambers, showing only a controlled dissolution and associated motion in the conditioned medium (see Fig. 3a and SI Movie 3). In presence of non-conditioned cell media CaCO_3_ Janus particles were stable (no difference in its shape or diameter was observed) even after 5 hours, only showing Brownian motion (see Fig. 3b).

**Figure 4:**
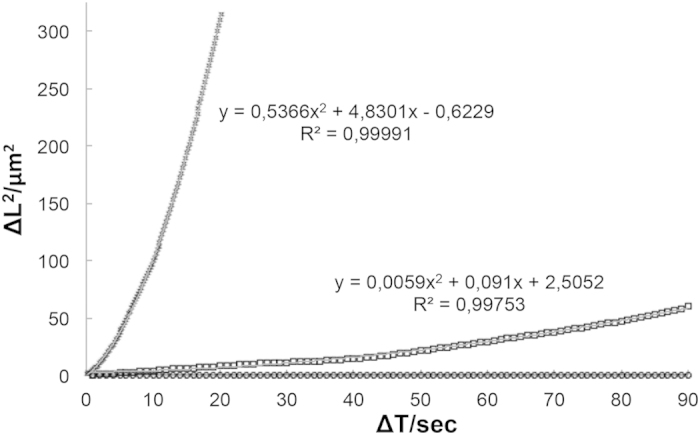
Statistics of CaCO_3_ Janus particles motion. Mean-squared displacements of particles at short interval of CaCO_3_ Janus particles at in acidic conditions (2.5:1.25 nM, Mannitol:dihydrogen citrate) (×track) and in conditioned cell media (□ track), and reference particle in conditioned cell media (○ track). Track× and □ in Fig. 4 present the corresponding fitting equation described by Dunderdale *et al.*^45^.

By working with complex cell medium, we did not expect diffusioosmotic flows to be as significant as in unsaturated acidic solutions. In order to be able to carefully evaluate the motion of Janus particles under such particular conditions, reference particles (Fluoro-Max, 10 μm green fluorescent polymer microspheres) were introduced in the system (see Movie S4). Our statistical analysis of the motors’ motion relied on the in-depth examination of nanopropulsion of particles reported by Dunderdale *et al*^45^. and were applied to both, controlled acidic conditions and the cell-conditioned media. Over short periods of time, our Janus particles revealed unidirectional propulsion with a ballistic trajectory under both acidic conditions (2.5:1.25 nM, Mannitol:dihydrogen citrate and conditioned cell media) but not in negative controls. These results are displayed in the characteristic curved mean-squared displacement vs time plot, while reference particles in the same system showed only Brownian motion (see Fig. 4). By applying the equation <ΔL>^2^ = 4*D*Δ*t* + v^2^Δ*t*^2^ 45 to the obtained mean-squared displacement vs time plot we observed that only the data obtained in acidic conditions fits the mentioned equation, showing that the apparent diffusion constant decreases in conditioned media when compared to known acidic conditions (2.5:1.25 nM Mannitol:dihydrogen citrate solution), as expected.

### Motion of CaCO_3_ Janus particles in tumour acidic microenvironments generated by Hela cells *in situ*

**Figure 5:**
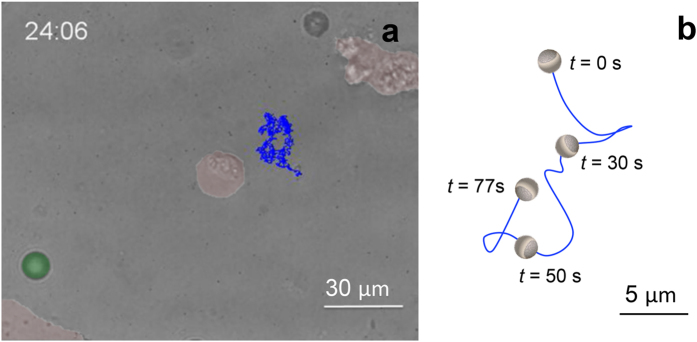
CaCO_3_ Janus particles motion in tumour acidic microenvironments. Tracking of CaCO_3_ Janus particles in tumor acidic microenvironments created *in situ* by HeLa cells incubated in non-buffered medium. For the sake of clarity, green fluorescent polymer microspheres used as reference particles are highlighted in green and H2B-mCherry HeLa cell nuclei are highlighted in red. Real time (min) in which the optical image was taken.

Once CaCO_3_ Janus particles showed an effective motion in conditioned cell media, the next logical step was checking their motion in an environment, where the acid producing HeLa cells were actually present in the petri dish during the experiment. This way we were able to observe that the dissolution and movement of particles happened as a consequence of the change of pH by the conversion of the initial cell media into conditioned, more acidic media *in vitro*. To this end, into the petri dish harbouring HeLa cells, Janus micromotors were added together with fluorescent reference polymer-based particles (see schematic in SI Figure S5C). Micromotors motion was evaluated showing a continuous and directional motion with a speed of 0.544 μm/s due to the lower acidic pH concentration in conditioned cell medium (see Movie S5 and Fig. 5). It should be noted that reference particles were conveniently used as artefacts to ensure the absence of flow during the experiment. Therefore, we were able to attribute the observed CaCO_3_ Janus particles motion to its controlled dissolution under the acidic conditions.

## Discussion

We have demonstrated the feasibility of using biocompatible micromotors moving in absence of any surfactants and external fuel addition, but in presence of fuel created *in situ* by HeLa cells. Our studies of CaCO_3_ Janus particle motors reveal dynamic, non-Brownian behaviour at nanomolar acidic concentrations of eight orders of magnitude lower than reported before^43^. This led us to apply them to a tumour cell system, where the acidic fuel was created *in situ* leading to slower, but continuous and effective motion. This simple but efficient motor configuration opens up future prospects in drug delivery, offering a unique rational on-board release in presence of acidified tumor microenvironments and several advantages associated with the motion of self-propelled entities such as chemotactic behavior^30^. The full potential will be unleashed in a next generation of Janus CaCO_3_ particles, which are downscaled to the nanometer size and loaded with drugs of interest.

## Methods

### CaCO_3_ Janus microparticles preparation

The synthesis was performed in a Elmasonic S10 ultrasonic bath (Elma Schmidbauer GmbH, Germany) by mixing at once 5 ml of CaCl_2_ (0.33 M) and 5 ml of Na_2_CO_3_ (0.33 M) during 15 min (both reagents were purchased from Sigma Aldrich). The mixture was rapidly centrifuged at 6000 rpm during 1.5 min, and washed 5 times with deionised water, being finally redispersed in 5 ml of deionized water. As to obtain a thin monolayer of the CaCO_3_ particles, glass substrate was cleaned with acetone and isopropanol to be further exposed to plasma cleaner during 30 sec. Due to the increased surface hydrophilic properties of the glass substrate the drop of the dispersed particles deposited onto was homogeneously dispersed, followed by a rapid soaking of the exceeding solution from one corner of the glass. Once the glass is dry, a layer of 0.8 nm of cobalt was sputtered onto the full surface, only covering half of the CaCO_3_ particles and therefore obtaining the desired non-symmetric Janus particles.

### Cell culture in non-buffered media

HeLa cells and NIH3T3 Fibroblasts were maintained in high glucose DMEM (SIGMA D-2429, non-pH buffered), supplemented with 10% Fetal Bovine Serum, 1% Glutamine and 1% Penicillin/Streptomycin. HeLa H2B-mCherry cells were a gift from Daniel Gerlich and showed the nucleus in red fluorescence, were kept as above but Puromycin was added to the culture medium every other day. Medium was changed every other day and cells were passaged once a week. For the particle movement in the presence of tumorous cell conditioned, acidic medium, 2 × 10^4^ HeLa cells (tumoural) or Fibroblasts (control) were seeded to cover one half of a petri dish with 35mm diameter. The experiments were conducted within 2 days. For experiments with the conditioned medium only, cells were seeded into a 25 cm^2^ cell culture bottle and allowed to acidify the medium. The medium was aspirated 4–5 days later and used for experiments.

### Equipment

Videos obtained in static conditions were recorded at 50 frames per second using Zeiss axioScope A1 optical microscope in bright-field, coupled with an ×40 objective. Regarding the experiments held for the evaluation of CaCO_3_ Janus particles motion in presence of conditioned acidic medium from tumour cells, videos were recorded at 10 frames per second using the inverted microscope Zeiss axioScope A1. In both cases, Phantom Miro eX2 high-speed camera from VisionResearch was used to record the videos in the above-mentioned conditions.

### Statistics

The acquired images and particles trajectories were recorded and processed by using MTrackJ plug-in from Fiji (image processing package, distribution of ImageJ). MSD evaluation was done in R (version 3.0.2, 32 bit), where mean squared displacement (MSD(τ)) is defined as the average squared Euclidian distance a CaCO_3_ Janus particle has covered after a certain time interval Δt and was calculated by using the equation: MSD(τ) = <[x(t+τ)-x(t)]^2^ +[y(t+τ)-y(t)]^2^>. Further data treatment done according to the studies reported by Dunderdale *et al.*^45^. Obtained mean-squared displacement vs time difference plot is fitted to the equation <ΔL>^2^ = 4*D*Δ*t* + v^2^Δ*t*^2^. Corresponding equation is depicted next to the MSD curve, where y is the <ΔL>^2^ and x is the Δ*t.*

## Additional Information

**How to cite this article**: Guix, M. *et al.* Carbonate-based Janus micromotors moving in ultra-light acidic environment generated by HeLa cells *in situ*. *Sci. Rep.* **6**, 21701; doi: 10.1038/srep21701 (2016).

## Supplementary Material

Supplementary Movie S1

Supplementary Movie S2

Supplementary Movie S3

Supplementary Movie S4

Supplementary Movie S5

Supplementary Information
