# Supplementary material for: Carbonate-based Janus micromotors moving in ultra-light acidic environment generated by HeLa cells in situ
Source: Sci Rep. 2016 Feb 24;6:21701. doi: 10.1038/srep21701 (PMC4764847; doi:10.1038/srep21701)
Supplement: Supplementary Information [file srep21701-s6.pdf]

Supporting Information

Carbonate-based Janus micromotors moving in ultra-light acidic environment generated by HeLa cells *in situ*

Maria Guix, Anne K. Meyer, Britta Koch, and Oliver G. Schmidt*

Supplementary Figures

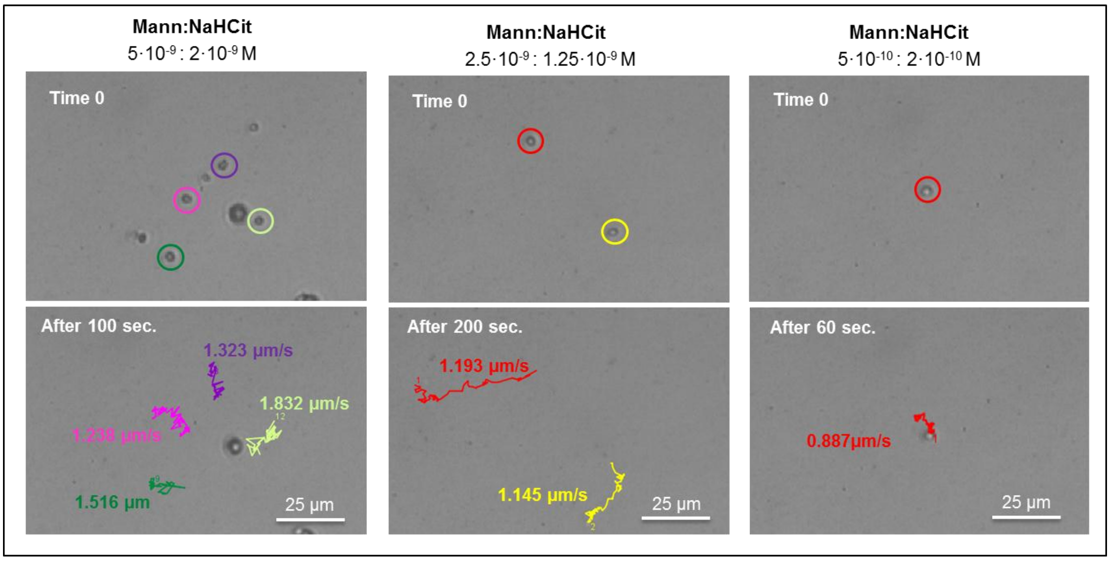

Figure S1. $CaCO_3$ Janus particles motion in static conditions. $CaCO_3$ Janus particles tracking under different acidic working conditions in parafilm platforms: 5:2 nM, 2.5:1.25 nM and 0.5:0.2 nM Mannitol:Sodium dihydrogen citrate concentration, at initial time and after 100 seconds, 200 seconds and 60 seconds, respectively. Scale bar, 25 µm.

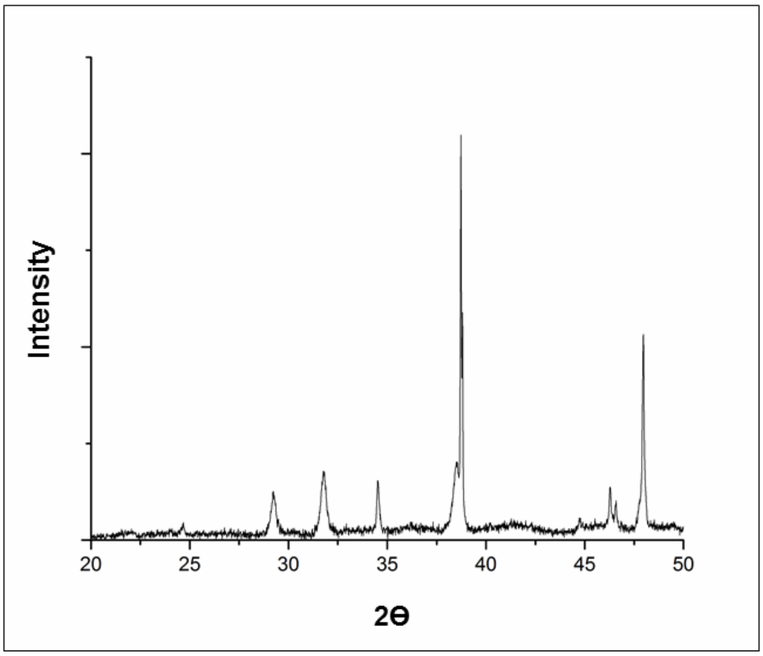

Figure S2. XRD characterization. XRD patterns correspond to $CaCO_3$ microparticles onto silicon wafer where a calcite-vaterite structure was revealed. The bands related to calcite structure were observed around 24.5 cm^{-1}, 29 cm^{-1}, 34.5 cm^{-1}, 39 cm^{-1} and the double band around 46 cm^{-1}. Regarding vaterite structure, the corresponding bands were located around 32 cm^{-1} and 48 cm^{-1}. It should be noted that the band, which would correspond to silicon can be found around 39 cm^{-1}.

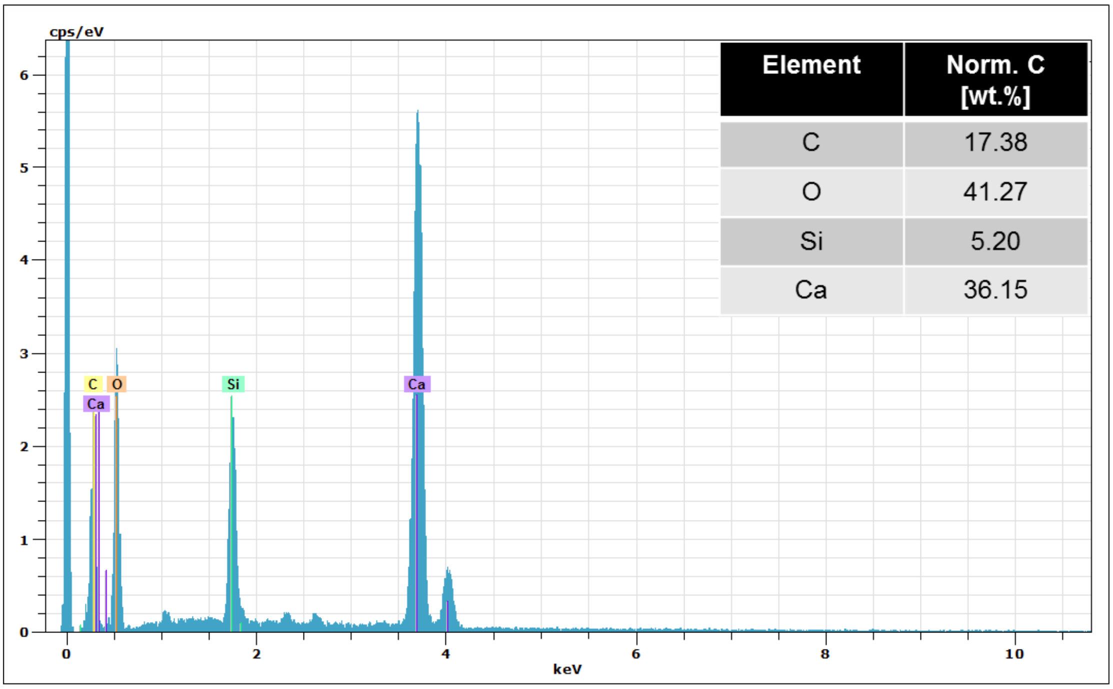

Element	Norm. C [wt.%]
C	17.38
O	41.27
Si	5.20
Ca	36.15

Figure S3. $CaCO_3$ particles composition. X-ray microanalysis of $CaCO_3$ Janus particles mounted onto a silicon wafer, using a Scanning Electron Microscope (SEM) Ultra Plus (Zeiss). Inset: Table showing the different elements present, with their corresponding weight percentage, of $CaCO_3$ Janus particles.

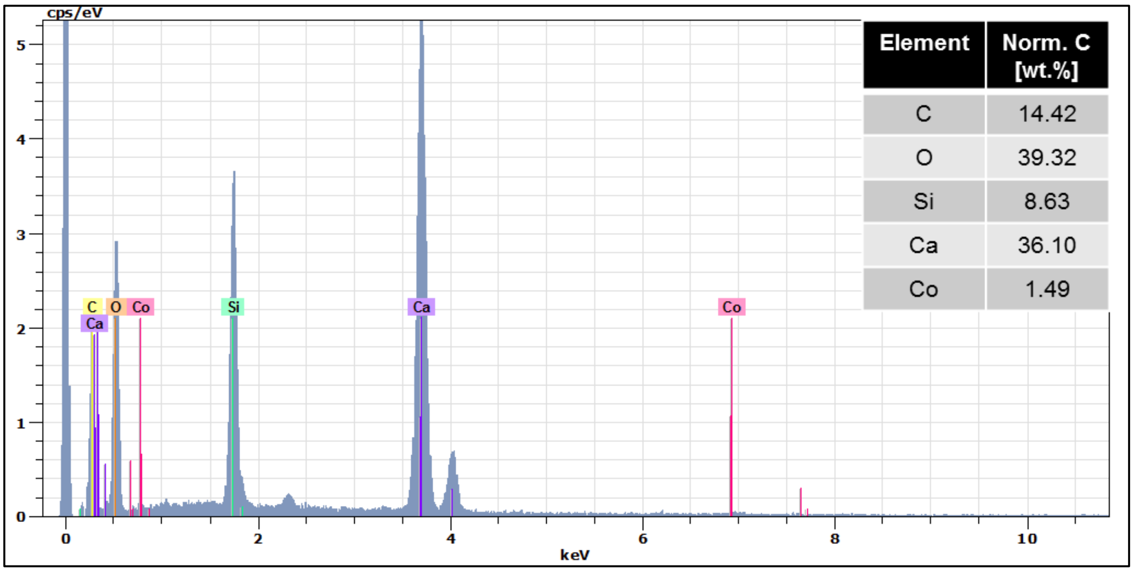

Element	Norm. C [wt.%]
C	14.42
O	39.32
Si	8.63
Ca	36.10
Co	1.49

Figure S4. $CaCO_3$ Janus particles composition. X-ray microanalysis of $CaCO_3$ Janus particles mounted onto a silicon wafer, using a Scanning Electron Microscope (SEM) Ultra

Plus (Zeiss). Inset: Table showing the different elements present, with their corresponding weight percentage, of $CaCO_3$ particles with a 0.8nm cobalt layer.

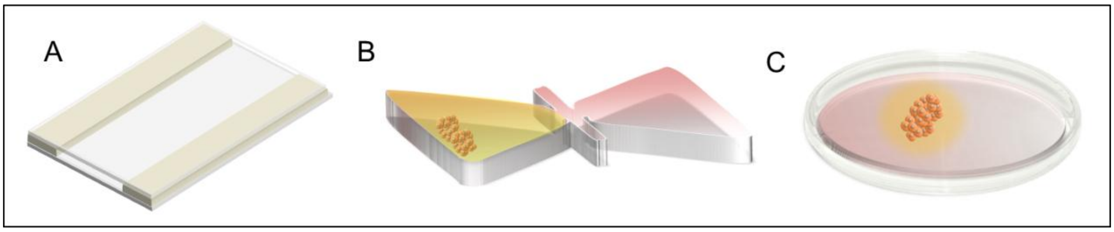

Figure S5: Schematic of different platforms used along all the experiments (real photo and schematic).

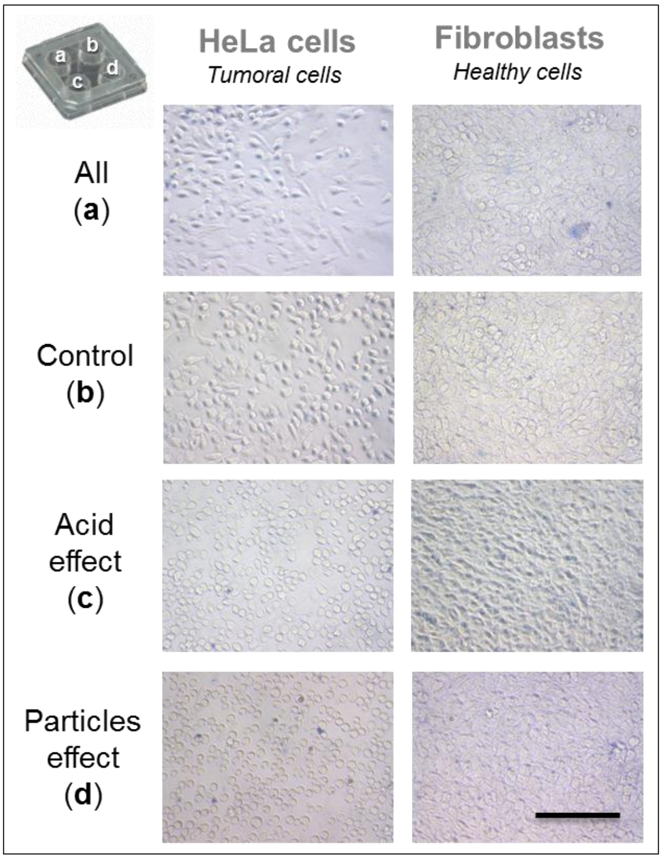

Figure S6. Viability test to evaluate acid and particles effect. Viability test of HeLa cells (tumor cells) and Fibroblast (healthy cells) performed by co-culturing them in the same petri dish with pH-buffered medium and incubation time of 6 hours in presence of acid mixture and $CaCO_3$ Janus particles (**a**); none of them (**b**); only with acidic mixture, 2.5:1.25 nM, Mannitol:dihydrogen citrate (**c**); and only with $CaCO_3$ Janus particles (**d**). Scale bar, 200 µm.

Supplementary videos

Movie S1: $CaCO_3$ Janus particles motion in paraffilm platforms at in presence in presence of 2.5:1.25 nM concentration of Mannitol:dihydrogen citrate.

Movie S2: $CaCO_3$ particles in presence of conditioned cell media.

Movie S3: $CaCO_3$ Janus particles in presence of conditioned cell media and non-buffered cell media.

Movie S4: $CaCO_3$ Janus particles in presence of conditioned cell media (associated statistical studies). Real time indicated in minutes.

Movie S5: $CaCO_3$ Janus particles motion in tumor acidic microenvironments *in situ* generated by Hela cells. Real time indicated in minutes.
